# Retraction: Identification of novel SCN5A single nucleotide variants in Brugada syndrome: a territory-wide study from Hong Kong

**DOI:** 10.3389/fphys.2024.1532081

**Published:** 2024-11-28

**Authors:** Frontiers Editorial Office

The journal retracts the 18 September 2020 article cited above.

Following publication, concerns were raised regarding the ethical approval for this study. An investigation was conducted by Hong Kong Hospital Authority Research Ethics Committee (REC). This committee confirmed that this study did not receive appropriate ethical approval.

An investigation was then conducted in accordance with Frontiers’ policies, and it was also confirmed that, contrary to the statement in the article, the study did not receive appropriate ethical approval. Lack of appropriate ethical approval is a breach of Frontiers’ guidelines and those of the Committee on Publication Ethics. As such, this article is being retracted.

The retraction was approved by the Chief Editors for Frontiers in Physiology and the Chief Executive Editor of Frontiers. The authors do not approve of the retraction.

